# Development of a Resveratrol Nanosuspension Using the Antisolvent Precipitation Method without Solvent Removal, Based on a Quality by Design (QbD) Approach

**DOI:** 10.3390/pharmaceutics11120688

**Published:** 2019-12-17

**Authors:** Do-Hoon Kuk, Eun-Sol Ha, Dong-Hyun Ha, Woo-Yong Sim, Seon-Kwang Lee, Ji-Su Jeong, Jeong-Soo Kim, In-hwan Baek, Heejun Park, Du Hyung Choi, Jin-Wook Yoo, Seong Hoon Jeong, Sung-Joo Hwang, Min-Soo Kim

**Affiliations:** 1 College of Pharmacy, Pusan National University, 63 Busandaehak-ro, Geumjeong-gu, Busan 46241, Korea; gdh7729@naver.com (D.-H.K.); edel@pusan.ac.kr (E.-S.H.); biz_magic@naver.com (D.-H.H.); popo923@pusan.ac.kr (W.-Y.S.); lsk7079@pusan.ac.kr (S.-K.L.); sui15@pusan.ac.kr (J.-S.J.); jinwook@pusan.ac.kr (J.-W.Y.); 2 Dong-A ST Co. Ltd., Giheung-gu, Yongin, Gyeonggi 446-905, Korea; ttung2nd@naver.com; 3 College of Pharmacy, Kyungsung University, 309, Suyeong-ro, Nam-gu, Busan 48434, Korea; baek@ks.ac.kr; 4 Department of Industrial and Physical Pharmacy, College of Pharmacy, Purdue University, 575 Stadium Mall Drive, West Lafayette, IN 47907, USA; pharmacy4336@gmail.com; 5 Department of Pharmaceutical Engineering, Inje University, Gyeongnam 621-749, Korea; choidh@inje.ac.kr; 6 College of Pharmacy, Dongguk University, Goyang 410-820, Korea; shjeong@dongguk.edu; 7 College of Pharmacy and Yonsei Institute of Pharmaceutical Sciences, Yonsei University, 85 Songdogwahak-ro, Yeonsu-gu, Incheon 21983, Korea; sjh11@yonsei.ac.kr

**Keywords:** resveratrol, nanosuspension, quality by design, optimization, bioavailability, dissolution

## Abstract

The purpose of this study was to develop a resveratrol nanosuspension with enhanced oral bioavailability, based on an understanding of the formulation and process parameters of nanosuspensions and using a quality by design (QbD) approach. Particularly, the antisolvent method, which requires no solvent removal and no heating, is newly applied to prepare resveratrol nanosuspension. To ensure the quality of the resveratrol nanosuspensions, a quality target product profile (QTPP) was defined. The particle size (z-average, d90), zeta potential, and drug content parameters affecting the QTPP were selected as critical quality attributes (CQAs). The optimum composition obtained using a 3-factor, 3-level Box–Behnken design was as follows: polyvinylpyrrolidone vinyl acetate (10 mg/mL), polyvinylpyrrolidone K12 (5 mg/mL), sodium lauryl sulfate (1 mg/mL), and diethylene glycol monoethyl ether (DEGEE, 5% *v*/*v*) at a resveratrol concentration of 5 mg/mL. The initial particle size (z-average) was 46.3 nm and the zeta potential was −38.02 mV. The robustness of the antisolvent process using the optimized composition conditions was ensured by a full factorial design. The dissolution rate of the optimized resveratrol nanosuspension was significantly greater than that of the resveratrol raw material. An in vivo pharmacokinetic study in rats showed that the area under the plasma concentration versus time curve (*AUC*_0–12h_) and the maximum plasma concentration (*C*_max_) respectively, than those of the resveratrol raw material. Therefore, the prepara values of the resveratrol nanosuspension were approximately 1.6- and 5.7-fold higher,tion of a resveratrol nanosuspension using the QbD approach may be an effective strategy for the development of a new dosage form of resveratrol, with enhanced oral bioavailability.

## 1. Introduction

Resveratrol, 3,5,4′-trihydroxystilbene, is a non-flavonoid polyphenolic antioxidant and it is produced by plants in response to injury or attack by bacteria and fungi. Resveratrol is found in a variety of dietary botanicals including grapes, peanuts, mulberries, cranberries, blueberries, and red wine [1]. It has been reported that resveratrol can afford health promotion in several chronic conditions such as aging, heart diseases, and cancers [2,3]. Resveratrol is a class II compound in the Biopharmaceutical Classification System (BCS) with high permeability (Log P = 3.1) and low aqueous solubility [2]. This property cause of its instability, poor water solubility, short biological half-life, and rapid metabolism and elimination, hence the therapeutic applications of resveratrol are very limited [1]. Numerous strategies have been evaluated to overcome these limitations of resveratrol, such as the use of polymeric nanoparticles [4,5], solid lipid nanoparticles [6,7], self-emulsifying drug delivery systems [8,9], nanoemulsions [10,11], liposomes [12,13], nanosuspensions [14,15], and nanofibers [16]. However, resveratrol can act as a reductant that is oxidized more readily than other components, hence its oxidative degradation can be accelerated by heating during the manufacturing process of final product. Therefore, the selection of a suitable process to avoid these negative factors is very important for the manufacturing of the quality ensured final product containing resveratrol.

Nanosuspensions are stabilized colloidal dispersions of nano-sized drug particles in the presence of a polymer, a surfactant, or both as stabilizers. Nanosuspensions can be used to enhance the solubility of drug with poor solubility in aqueous or lipid solvents [17]. The nano-sized particles in nanosuspensions provide a large surface area, thus result in increased solubility and dissolution rate of poorly soluble drugs. Through this mechanism, nanosuspension formulation of BCS Class II and IV compounds can exhibit improved bioavailability, fast action and other desirable biopharmaceutical effects [17,18]. Technologies for the preparation of nanosuspension are roughly divided into two ways, top-down and bottom-up methods. The top-down methods reduce the drug particle size without organic solvent using techniques such as milling (jet mill and ball mill) and high-pressure homogenization. However, sometimes these top-down methods are difficult to apply to thermolabile materials because they are high-energy processes and thus, they generate heat. In addition, there are limits to the production of nanoparticles below submicron. Furthermore, a large amount of energy can produce amorphous particles and deform crystals [19,20]. The bottom-up method uses the particle precipitation from a saturated or unsaturated drug solution. The bottom-up methods include various techniques, such as solvent evaporation, supercritical fluid, antisolvent precipitation, and chemical precipitation. These methods require relatively small energy compared to top-down methods, but if harmful organic solvents are used, additional processes such as heating and freeze-drying must be required to remove them in the final formulation [17]. When applying the most widely used the antisolvent method, it is important to control the residual solvent and particle growth. Inappropriate control of particle growth is a consequence of an incomplete understanding of the formulation and manufacturing processes. Therefore, there has been a need for development of robust processes that do not involve additional harsh processes for organic solvent removal to prepare a nanosuspension [21]. These processes must be understood using scientific and systematic methods to successfully develop an efficient manufacturing method to produce the final drug product with the desired best quality. From this point of view, the quality by design (QbD) approach should be applied to the development of the nanosuspension at the formulation step as well as the preparation process.

Over the years, the use of QbD in the pharmaceutical sector has been described in the publications, ICH (International Council for Harmonization of Technical Requirements for Pharmaceuticals for Human Use) Q8 (pharmaceutical development), ICH Q9 (quality risk management), and ICH Q10 (pharmaceutical quality system). These provide a high-level of guidance regarding the scope and definition of QbD, as it applies to the pharmaceutical industry [22]. In accordance with the ICH Q8 guideline, QbD is defined as a systematic approach to development that begins with predefined objectives. In addition, it emphasizes understanding of the overall product control, as well as the process control, based on science and quality risk management [23]. To implement QbD, the quality target product profile (QTPP) and critical quality attributes (CQAs) must be defined. Critical material attributes and critical process parameters (CPPs) that affect CQAs, based on risk assessments (RAs) and prior knowledge, must be identified. A design space is established after design of experiment and risk analyses [17]. A key goal in product design and understanding is the development of robust products that can deliver the desired QTPP during the product lifecycle. Products manufactured using the QbD approach have improved stability and lower production costs, increased patient efficacy, and minimal side effects.

**Figure 1:**
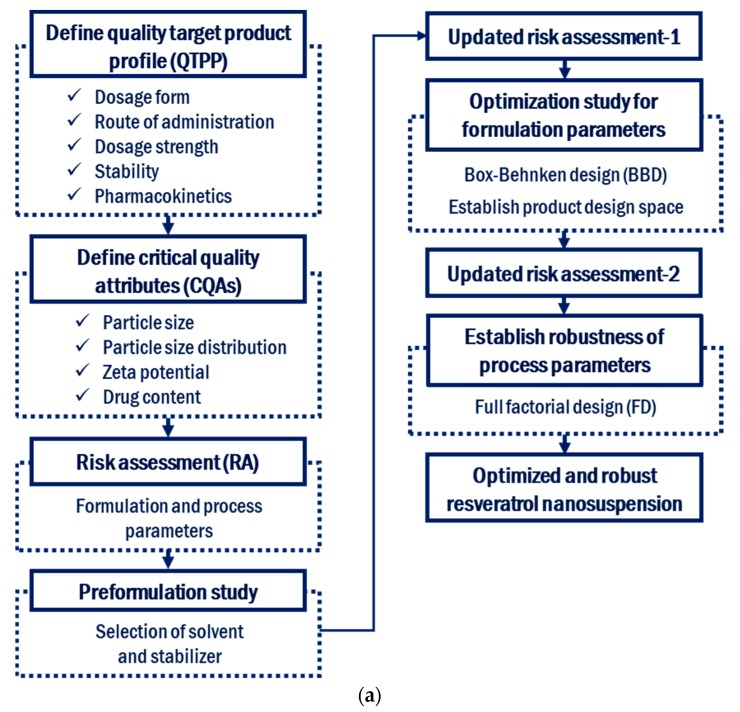

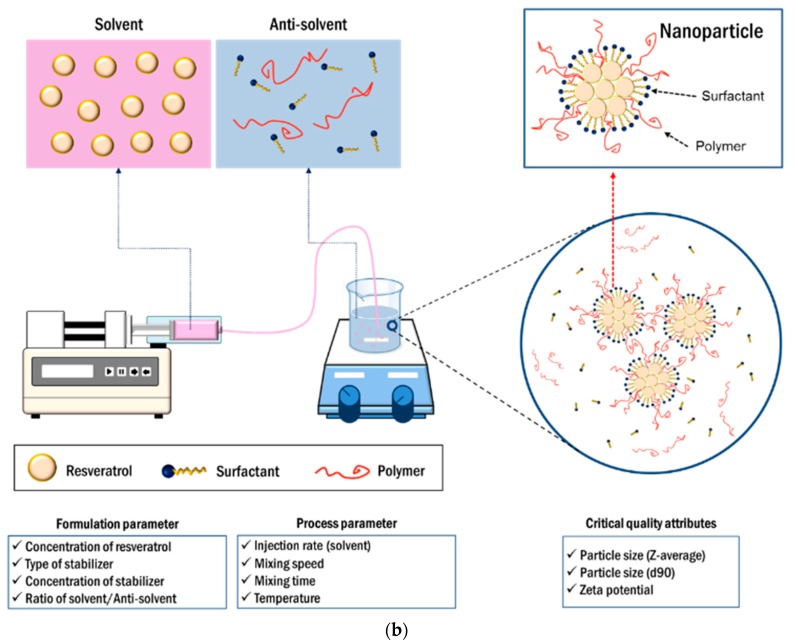
Development flow (**a**) and manufacturing process (**b**) of resveratrol nanosuspension based on the quality by design approach.

Currently, the research, development, manufacturing, storage, and clinical process development of nanosuspensions are in their infancy, and therefore, it is useful and necessary to apply QbD to this field. The major barriers to the manufacturing and clinical application of nanosuspensions include the destabilization of structures and an incomplete understanding of the manufacturing process. The purpose of this study was to develop a resveratrol nanosuspension with enhanced oral bioavailability using the antisolvent method without solvent removal, based on the QbD approach. To the best of our knowledge, there is no report about applying this kind of antisolvent technique to resveratrol for the preparation of nanosuspension. Figure 1 shows the flow of nanosuspension development based on QbD, which is used in this study. The QTPP, CQAs, and CPPs of the resveratrol nanosuspension were defined. RA was performed to identify the formulation and process parameters that affect the CQAs. Among the critical formulation parameters identified in the RA, appropriate formulation parameters were selected in a preliminary experiment. The selected formulation parameters were optimized using a 3-factor, 3-level Box–Behnken design, after which an updated RA was performed. A full factorial design was used to establish the robustness of the process parameters under the optimized composition conditions. In addition, in vivo pharmacokinetic studies were performed to compare the oral bioavailability of the resveratrol nanosuspension with that of the resveratrol raw material. Finally, the long-term stability of the resveratrol nanosuspension was evaluated.

## 2. Materials and Methods 

### 2.1. Materials

Resveratrol (99.0% purity, *trans*-form) was purchased from Kukjeon Pharm Co., Ltd. (Gyeonggi, Korea). Carbamazepine (99.0% purity) and sodium lauryl sulfate (SLS) were purchased from Sigma-Aldrich Co. (St Louis, MO, USA). Diethylene glycol monoethyl ether (DEGEE, Transcutol^®^ HP, Gattefossè, Saint-Priest, France) was obtained from Masung & Co., Ltd. (Seoul, Korea). Polyvinylpyrrolidone (PVP K12, K17, K25, and K30) and polyvinylpyrrolidone vinyl acetate VA64 (PVP VA64) were obtained from BASF Co., Ltd. (Kollidon^®^, Ludwigshafen, Germany). Hydroxypropyl cellulose polymers (HPC-L, HPC-SL, and HPC-SSL) were purchased from Nippon Soda Co., Ltd. (Tokyo, Japan). Hydroxypropyl methyl cellulose (HPMC, Hypromellose 3 cp, 6 cp, and 15 cp) was obtained from Shin-Etsu Chemical Co., Ltd. (Tokyo, Japan). Acetone (99.8% purity), 1-butanol (99.5% purity), and ethyl acetate (99.5% purity) were purchased from Daejung Chemical & Metals Co., Ltd. (Gyeonggi, Korea). Dichloromethane was purchased from Junsei Chemical Co., Ltd. (Tokyo, Japan). High-performance liquid chromatography (HPLC)-grade methanol, ethanol, acetonitrile, 1-propanol, and 2-propanol were purchased from Honeywell Burdick & Jackson (Morristown, NJ, USA).

### 2.2. Definition of the Quality Target Product Profile and Critical Quality Attributes

The QTPP is a prospective summary of the characteristics of a drug product that will ideally be achieved to ensure the desired quality, considering its safety and efficacy. QTPP forms the basis of design for the development of the product [23]. In this study, the QTPP was defined to produce a resveratrol nanosuspension with high stability and suitable oral bioavailability. A CQA is a physical, chemical, biological, or microbiological property or characteristic of an output material, including a finished drug product, which should meet the predefined requirements to ensure the desired product quality. CQAs are generally associated with the drug substance, excipients, intermediates (in-process materials), and drug products. The QTPP and CQA elements for the resveratrol nanosuspension, with targets and justifications, are defined in Table 1. 

### 2.3. Initial Risk Assessment for Formulation and Process Parameters

The purpose of the RA is to identify high-risk formulations and process parameters that can affect the CQAs of the drug product. RA is used to prioritize analyses by determining which parameters are important and which are not [22]. An initial RA was performed to identify the parameters that affect the CQAs of the resveratrol nanosuspension and to set risk levels. The initial RA is summarized in Table 2 and the details are shown in Table S1.

### 2.4. Preformulation of the Resveratrol Nanosuspension

#### 2.4.1. Solubility of Resveratrol in Various Solvents

The solubility of resveratrol was measured in various organic solvents. Excess amounts of resveratrol were added to a vial containing 10 mL of the organic solvent. Each experiment was performed in triplicate. Samples were mixed for 5 min by vortexing, followed by sonication for 1 h in an ultrasonic bath (model 5800; Branson, Danbury, CT, USA). The vials were placed in a shaking water bath (BS-21; Jeiotech Co Ltd., Daejeon, Korea) at 25 °C for 24 h. The resulting saturated solutions were filtered using a 0.45 µm regenerated cellulose (RC) syringe filter and transferred into a volumetric flask, followed by dilution with methanol. The concentration of resveratrol was then determined using a Shimadzu HPLC system with an SPD-20A ultraviolet-visible detector, a CBM-20A communication bus module, a SIL-20AC autosampler, an LC-20AT liquid chromatograph, a DGU-20A 5R degassing unit, and a CTO-20A column oven (Shimadzu, Tokyo, Japan). Chromatographic separation was performed using a Gemini C18 reversed-phase column (Phenomenex, Torrance, CA, USA; 150 mm × 4.6 mm, 5 μm). The injection volume was 10 μL. The mobile phase was composed of water and acetonitrile (60:40, *v*/*v*), with a flow rate of 0.8 mL/min at 30 °C. Detection was performed at a wavelength of 303 nm. A calibration curve was prepared by measuring the peak areas of known resveratrol standards in the concentration range of 1–100 μg/mL.

#### 2.4.2. Inhibitory Effect of Polymers on Resveratrol Precipitation

The inhibitory effect of polymers on the precipitation of resveratrol was measured using a USP rotating paddle apparatus (Electrolab, Mumbai, India) at 37 °C and 50 rpm, in 500 mL of distilled water (containing the polymers at 0.5% (*w*/*v*) concentrations). Samples (3 mL) were removed at the specified time points, filtered using a 0.45 μm RC syringe filter, and then diluted with methanol. The resveratrol concentration was then determined by HPLC.

### 2.5. Preparation of Resveratrol Nanosuspensions

Resveratrol nanosuspensions were produced using the antisolvent precipitation technique. Briefly, resveratrol was dissolved in transcutol HP. Meanwhile, an aqueous solution was prepared by dispersing the required concentration of polymers and surfactants in distilled water. The transcutol HP-resveratrol solution was rapidly added to the aqueous solution as the solvent, with magnetic stirring at 750 rpm. A blue-colored suspension immediately appeared and a resveratrol nanosuspension was prepared by mixing.

### 2.6. Design of Experiments for the Preparation of Resveratrol Nanosuspensions

#### 2.6.1. Box–Behnken Design for the Optimization of Formulation Parameters

The Box–Behnken design and response surface methodology were used to optimize the composition of the resveratrol nanosuspension. In this design, the experimental region is assumed to be a cube. The experiment is performed at the point corresponding to the midpoint of each axis and a repeated experiment is performed at the center of a multidimensional cube [25]. A total of 17 experiments were performed at three levels, with three factors. The central point was replicated five times. The formulation parameters and parameter levels were selected based on preliminary studies. PVP VA64 (X_1_), PVP K12 (X_2_), and SLS (X_3_) were selected as the formulation parameters (factors) at three levels (−1, 0, and +1). The particle size (z-average, d90) and zeta potential (ζ) were selected as responses. The formulation parameters and responses are shown in Table 3. The experimental design was generated and evaluated using Design Expert^®^ 11.0 (Stat-Ease, Inc. Minneapolis, MN, USA). A Monte Carlo simulation for risk analysis was carried out using MODDE 12 software (Umetrics Inc., Umeå, Sweden). The predicted response, Y, was calculated using the non-liner quadratic model equation as follows.

$$Y=\beta_{0}+\beta_{1}X_{1}+\beta_{2}X_{2}+\beta_{3}X_{3}+\beta_{4}X_{1}X_{2}+\beta_{5}X_{2}X_{3}+\beta_{6}X_{1}X_{3}+\beta_{7}X_{1}{}^{2}+\;\beta_{8}X_{2}{}^{2}+\beta_{9}X_{3}{}^{2},$$

where Y is the response, β_0_ is the intercept, and β_1_ to β_9_ are the regression coefficients. X_1_, X_2_, and X_3_ are the individual effects; X_1_X_2_, X_1_X_3_, and X_2_X_3_ are the interaction effects; and X_12_, X_22_, and X_32_ are the quadratic effects.

#### 2.6.2. Full Factorial Design to Establish the Robustness of the Process Parameters

In this study, a full factorial design was used to ensure the robustness of the process for the optimized composition. A total of nine experiments were performed for three factors at two levels and the central point was run once. The injection rate (X_1_; 0.5, 1.5 mg/mL), temperature (X_2_; 20, 30 °C), and mixing speed (X_3_; 500, 1000 rpm) were selected as the process parameters at two levels and the particle size (z-average, d90) and zeta potential (ζ) were used as responses.

### 2.7. Characterization of the Resveratrol Nanosuspension

#### 2.7.1. Measurement of Drug Content (%)

A certain amount of sample was transferred into a volumetric flask, followed by dilution to suitable concentration with methanol. Then, diluted sample was injected to HPLC system and the concentration of resveratrol was then determined as same method mentioned above. The drug content (%) was calculated by dividing the theoretical concentration by the measured concentration and multiplying by 100.

#### 2.7.2. Measurement of Particle Size and Zeta Potential

To characterize the particle surfaces, the particle size distribution and the zeta potential of the resveratrol nanosuspension were measured using a dynamic light scattering (DLS) instrument (ELSZ-1000; Photal Otsuka Electronics, Osaka, Japan) at 25 °C. Particle size was measured in triplicate and expressed as the z-average diameter. DLS was employed to determine the particle size and its distribution of the nanoparticles in suspension [26]. The zeta potential is a measure of the electric charge at the surface of the particles and is used to indicate the physical stability of colloidal systems. The zeta potential was measured using the laser Doppler method.

#### 2.7.3. Morphological Characterization

Morphological analysis was performed using a transmission electron microscope (TEM; H-7600; Hitachi, Tokyo, Japan) at 80 kV. The nanosuspension was dropped onto a carbon film-coated copper grid and then dried under a fume hood at room temperature for 24 h.

#### 2.7.4. In Vitro Dissolution Study

Dissolution was performed using a United States Pharmacopeia (USP) dissolution apparatus II (paddle method; Electrolab) at 37 ± 0.5 °C in 900 mL of distilled water (*C*_s_ = 55.3 μg/mL, *C* = *C*_s_), with a paddle speed of 50 rpm. The resveratrol nanosuspension (5 mg/mL, 10 mL) and resveratrol raw material (50 mg) were placed onto the dissolution vessel. Samples (5 mL) were withdrawn at different time intervals and replaced with fresh dissolution medium maintained at 37 ± 0.5 °C, to maintain a constant volume. The samples were immediately filtered using a 0.45 μm RC syringe filter and then diluted with methanol. The concentration of resveratrol was then determined by HPLC. Undissolved nanoparticles within the filtered sample were evaluated by DLS measurements.

### 2.8. Pharmacokinetic Study in Rats

The animal study protocol was in compliance with the institutional guidelines for the care and use of laboratory animals and was approved by the ethics committee of Kyungsung University (No. 17-003A, Feb. 22, 2017). Eight male Sprague–Dawley (SD) rats (200 ± 10 g; Orient Bio Inc., Seongnam, Korea) were divided into two treatment groups of four rats each. Prior to the study, the rats were fasted for 18 h. The two experimental groups received either the resveratrol raw material or the resveratrol nanosuspension, at resveratrol doses of 20 mg/kg, by oral administration. The nanosuspensions were prepared 1 week before oral administration. The resveratrol raw material was dispersed in 1 mL of water immediately prior to oral dosing. Blood samples (approximately 0.25 mL) were collected in heparinized tubes from the jugular vein of the treated rats at 0.25, 0.5, 0.75, 1, 1.5, 2, 4, 6, 8, and 12 h after dosing. Blood samples were centrifuged at 12,000× *g* for 10 min at 4 °C. The resulting plasma was transferred to individual centrifuge tubes and stored at −70 °C. Stock solution of resveratrol (1 mg/mL) and the internal standard, carbamazepine (1 mg/mL), were prepared in methanol. Standard working solutions of resveratrol (50 μg/mL) and carbamazepine (50 μg/mL) in a mixture of water and methanol (1:1) were freshly prepared before the experiments. The standard working solution of resveratrol (50 μg/mL) was serially diluted with a water-methanol (1:1) mixture and known amounts (10 μL) of the solutions were spiked into the blank plasma (100 μL) followed by the addition of 5 μL of the internal standard working solution (50 μg/mL). The plasma sample was then extracted by liquid–liquid extraction according to the protocol described below. Seven standard calibration solutions of resveratrol were prepared to obtain final concentrations ranging from 5 to 5000 ng/mL. Resveratrol and carbamazepine (internal standard) were extracted from the plasma by liquid–liquid extraction. Briefly, 40 μL of phosphate-buffered saline (30 mM, pH 6) was added to the plasma sample in a clean 2 mL centrifuge tube and the content was mixed for another 15 s by vortexing. Finally, ethyl acetate (300 μL) was added and mixed for 30 s. After ethyl acetate extraction, the sample was centrifuged at 8000 rpm for 10 min and the upper organic layer was carefully transferred to a clean tube. The extraction procedure was repeated another two times and the combined organic layers were evaporated to dryness under nitrogen gas at 35 °C using a heating block (Eyela MG-2200, Tokyo, Japan). The residue was reconstituted with 75 μL of the mobile phase and centrifuged for 10 min at 13,000 rpm. The supernatant was then transferred into a glass insert that was pre-installed in an auto-sampler vial. During each assay, 20 μL of the supernatant was injected to the HPLC system. The concentration of resveratrol was determined by HPLC, using a previously reported analytical method [27]. All procedures were performed away from direct light. Chromatographic separation was achieved with a reversed-phase HPLC column (Phenomenex, Torrance, CA, USA; EVO 5 μm C18, 250 mm × 4.6 mm), which was protected by a guard column (Phenomenex, Torrance, CA, USA; Gemini C18, 4 mm × 3.0 mm). The assay was performed by the isocratic delivery of the mobile phase, consisting of acetonitrile and 30 mM phosphate-buffered saline (pH 7.0; 30:70 *v*/*v*), at a flow rate of 1 mL/min at 35 °C. The mobile phase was filtered through a 0.2 mm nylon membrane and degassed by sonication for 20 min at 25 °C prior to use. Detection was performed by measuring absorbance at 306 nm.

### 2.9. Stability Study

The stability study for the resveratrol nanosuspension was performed for a period of 6 months under long-term storage conditions (25 ± 5 °C, 60% RH). At 1-month intervals, the resveratrol nanosuspension was characterized with respect to particle size and drug content.

### 2.10. Statistical Analysis

To evaluate the statistical significance of differences between two groups, Student’s *t*-test was carried out using SPSS 25.0 software (IBM SPSS Statistics, Chicago, IL, USA).

## 3. Results and Discussion

### 3.1. Preformulation of the Resveratrol Nanosuspension

#### 3.1.1. Solubility of Resveratrol in Solvents

**Figure 2:**
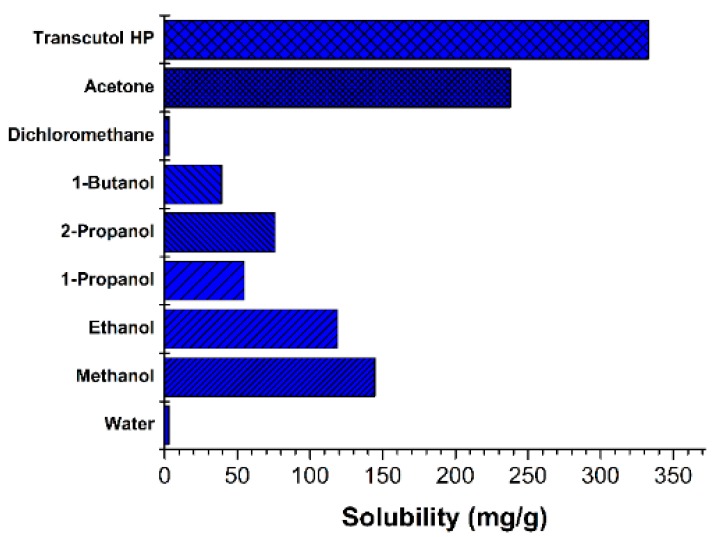
Solubility of resveratrol in various solvents at 25 °C.

The solubility of resveratrol was measured in various solvents (Figure 2). Its solubility in transcutol HP and acetone was 332.9 mg/g and 238.0 mg/g, respectively. Transcutol HP had low toxicity and a strong solubilization effect. Additionally, transcutol HP is advantageous in that it does not require the removal of the solvent during the nanosuspension manufacturing process, since it can be ingested in small quantities. Transcutol HP has been used as a solvent in many products, including medicines, (e.g., Lysanxia, Pilosuryl, and Urosiphon) cosmetics, and food additives [28,29]. Therefore, transcutol HP was selected as the solvent in this study.

#### 3.1.2. Inhibitory Effect of Stabilizers on Resveratrol Precipitation

**Figure 3:**
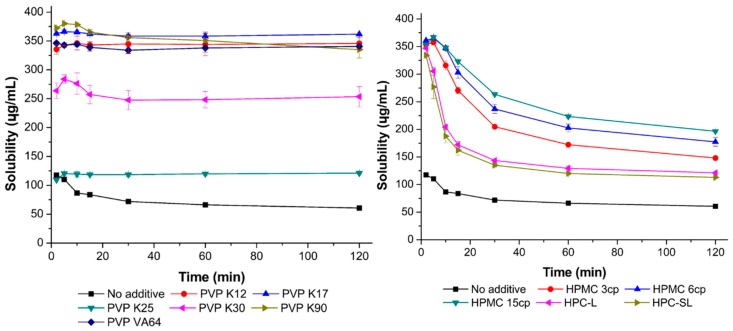
Inhibitory effects of PVP, HPC, and HPMC on resveratrol precipitation.

As shown in Figure 3, the inhibitory effects of PVP VA64, PVP, HPMC, and HPC on resveratrol precipitation were determined. In the absence of a stabilizer, resveratrol precipitated rapidly. PVP and PVP VA64 showed superior precipitation inhibition abilities compared with HPMC and HPC. In the case of PVP K12, PVP K17, and PVP VA64, resveratrol concentration greater than 330 μg/mL was maintained for 120 min. In contrast, when HPMC and HPC were used, particle agglomeration occurred gradually over time. Therefore, in the nanosuspension preparation, PVP and PVP VA64 controlled particle growth more effectively than HPMC and HPC.

#### 3.1.3. Screening for Stabilizers

One of the major problems with nanosuspensions is the difference in saturation solubility and concentration gradient, which can lead to Ostwald ripening [26]. This phenomenon can be controlled using various additives. The main function of a stabilizer is to prevent Ostwald ripening and the agglomeration of the nanosuspension, by providing a steric or ionic barrier to form a physically stable formulation [24]. The selection of the type and amount of stabilizer is very important. In this study, particle stabilization was determined in the presence of a polymer, a surfactant, or both, to select the appropriate stabilizer. In the initial screening study, the formulation and process conditions (injection rate: 1 mL/min, mixing speed: 750 rpm, and temperature: 25 °C) were selected from previously reported studies [29,30]. The resveratrol concentration in transcutol HP and the solvent/antisolvent ratio were selected as 100 mg/mL and 1/19, respectively, from the solubility behavior of resveratrol in Transcutol HP and water at different temperatures [30].

The stabilization abilities of HPMC and PVP, with various viscosities, and PVP VA64 were determined at various concentrations (see Supplementary Materials, Table S2) by visually observing particle aggregation during the manufacturing process. PVP, PVP VA64, and HPMC did not sufficiently inhibit particle growth. In addition, the stabilization abilities of the combinations of PVP VA64/PVP and HPMC 6 cp/PVP were determined at various concentrations (see Supplementary Materials, Table S3). PVP VA64/PVP and HPMC 6 cp/PVP combinations did not sufficiently inhibit particle growth. Furthermore, the stabilization abilities of the combinations of PVP VA64/PVP/SLS and HPMC 6 cp/PVP/SLS were determined at various concentrations (see Supplementary Materials, Table S4). The PVP VA64/PVP K12/SLS (1.0%/0.5%/0.1%, *w*/*v*) combination had the greatest stabilizing ability, with a particle size of 46.5 nm. Although the HPMC 6 cp/PVP/SLS combination resulted in a particle size of 210.4–236.8 nm, particle aggregation was observed during the manufacturing process. Therefore, PVP VA64, PVP K12, and SLS were selected as the stabilizers. The manufacturing process of nanosuspensions is thermodynamically unstable. Thus, stabilizers such as polymers and surfactants are essential for maintaining a physically stable state. The nonionic polymer, PVP, may be fixed on the surface of the drug particle to occupy adsorption sites and prevent drug molecules from binding to a crystal lattice in solution [31], thereby providing a mechanical barrier to crystallization. However, when the polymer concentration is insufficient, crystals can grow rapidly and aggregate. If the concentration of the polymer is continuously increased, the particle size increases due to the presence of a thick layer on the particle surface and diffusion between the solvent and the anti-solvent is suppressed during precipitation. Additionally, as the concentration of the polymer increases, the osmotic pressure increases, causing an increase in the attraction between colloidal particles [32,33]. This leads to particle growth. The surfactant is adsorbed at the solid–liquid interface, reducing the surface tension of the interface and increasing the nucleation rate, resulting in an initial decrease in particle size. In addition, the adsorption of surfactants reduces hydrophobic interactions and coagulation, making resveratrol particles less hydrophobic and reducing particle growth [34,35]. In particular, when SLS, which is an anionic surfactant, is adsorbed on the particle surface, the particle surface is negatively charged. This increases the repulsive force between the particles to increase the energy barrier and thereby, prevent particle growth and aggregation [36]. As a result, the addition of an appropriate concentration of a stabilizing agent can lower the excessively high surface energy of the resulting nanoparticles.

### 3.2. Optimization Study of the Resveratrol Nanosuspension

For development of a 5 mg/mL resveratrol nanosuspension, the stabilizer type (PVP VA64, PVP K12, and SLS), resveratrol concentration in Transcutol HP (100 mg/mL), and the ratio of solvent/antisolvent (1/19) were selected based on the results of preliminary experiments (see Supplementary Materials, Table S5), thereby reducing the risk level to low. The nanosuspensions were prepared using the optimal manufacturing process conditions (injection rate: 1 mL/min, mixing speed: 750 rpm, and temperature: 25 °C) identified in preliminary experiments. However, the risk level for this process was only reduced to medium, because additional experimentation was required to establish the robustness of the process parameters. RA was performed based on experimental data from preliminary experiments and these results are summarized in Table 2 (see Supplementary Materials, Table S6).

#### 3.2.1. Box–Behnken Design for the Optimization of Formulation Parameters

**Figure 4:**
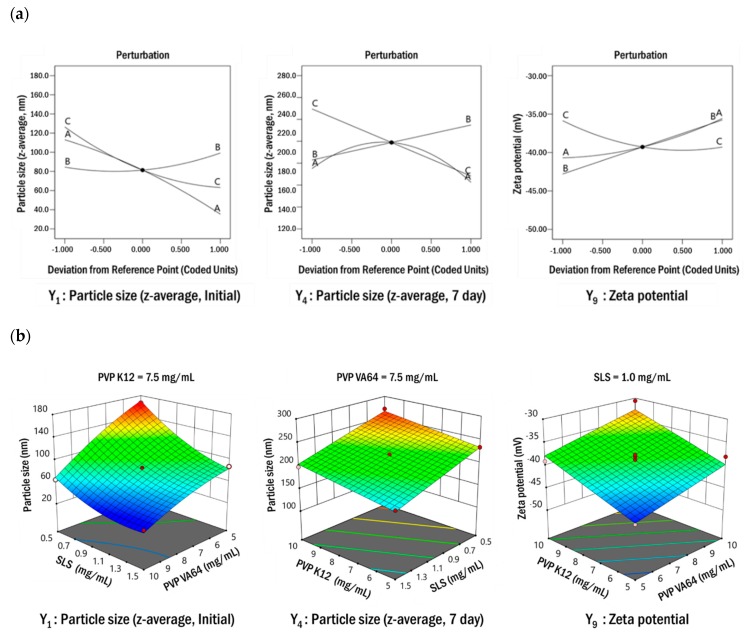
Perturbation plots (**a**) and response surface plots (**b**) showing the effects of various formulation parameters on the responses Y_1_, Y_4_, and Y_9_.

To determine the optimal resveratrol nanosuspension composition, 17 resveratrol nanosuspensions were prepared with varying concentrations of PVP VA64 (X_1_), PVP K12 (X_2_), and SLS (X_3_), according to the matrix generated by the Box–Behnken design. The results obtained for particle size and zeta potential are summarized in Table 4. For all prepared nanosuspensions, the particle size (z-average, initial) was less than 200 nm, and the particles grew over time. The zeta potential of the prepared nanosuspensions was in the range of −30.63 to −45.12 mV, which satisfied the target values of the response (>±20 mV). A regression analysis was used to assess the functional relationship between the formulation parameters and responses and thereby and to identify a particular pattern and the most appropriate regression function. The coefficients in the regression analysis were estimated based on the least squares method. The main effects, interactions, and curvature effects of each factor were obtained using an analysis of variance (ANOVA). In the ANOVA, data were analyzed based on the *F*-value, to determine which factors were significantly related to the response. Only responses with a *p*-value < 0.05 were considered statistically significant. A lack-of-fit test was used to determine whether the mean value of repeated measurements was appropriate for the estimated model. In this test, a *p*-value > 0.05 indicated conformity. Table 5 shows the results of regression analyses and ANOVA for the responses. The *p*-values for all models were <0.05, indicating significance. The *p*-values of the lack-of-fit tests exceeded 0.05, indicating a lack of significance. In the models for Y_1_–Y_9_, the regression coefficients (*R*^2^) were in the range of 0.9959–0.8629. A regression coefficient of 0.8 or higher is usually considered to indicate a good model. Positive coefficients indicate an advantageous effect in the optimization analysis, while negative values indicate an inverse relationship between the factor and the response. As shown in Table 5, the concentration of PVP VA64 (X_1_) and the concentration of SLS (X_3_) had negative effects on particle size (Y_1_–Y_8_), while the concentration of PVP K12 (X_2_) had a positive effect. These results indicated that the particle size increased as the concentration of PVP K12 (X_2_) increased or the concentrations of PVP VA64 (X_1_) and SLS (X_13_) decreased. In particular, the effect of PVP K12 concentration (X_2_) on particle size (z-average, Y_1_–Y_8_) increased with time. The PVP VA64 (X_1_) and SLS (X_3_) concentrations had a greater influence on particle size than the PVP K12 concentration (X_2_) at the initial stage of production. However, in the process of maintaining the particle size, all formulation parameters had a substantial influence. For zeta potentials (Y_9_) with negative values, PVP VA64 (X_1_) and PVP K12 (X_2_) concentration had positive effects and SLS concentration (X_3_) had a negative effect. PVP VA64 and PVP K12 are non-ionic polymers and SLS is an anionic surfactant. The charge on the particle surface becomes negative as the concentration of SLS increases or the concentration of the polymer decreases. When the surface is negatively charged, an ion barrier forms between the particles to inhibit particle growth. For the response surface design, perturbation plots (Figure 4a) show how the response changed as each parameter moved from the chosen value, with all other parameters held constant at zero. SLS concentration had the greatest effect on particle size. The effect of PVP VA64 concentration on particle size decreased with time; however, the effect of PVP K12 concentration increased. A similar tendency was observed for the response variables, Y_5_–Y_8_. All parameters had a significant effect on zeta potential. As shown in Figure 4b, the relationship between the formulation parameters and response variables was determined by the response surface plot. To create a response surface plot, only two formulation parameters can be represented at a time; accordingly, one formulation parameter must always be fixed. Figure 4b shows that the particle size decreased as PVP VA64 and SLS concentrations increased. After 7 days, the particle size decreased as PVP K12 concentration decreased or SLS concentration increased. A similar tendency was observed for the response variables, Y_5_–Y_8_. Zeta potentials with negative values decreased as PVP VA64 and PVP K12 concentrations increased. This was consistent with the negative contribution of SLS and the positive contribution of PVP VA64 and PVP K12 shown in Table 5 and Figure 4.

#### 3.2.2. Establishment of the Design Space

**Figure 5:**
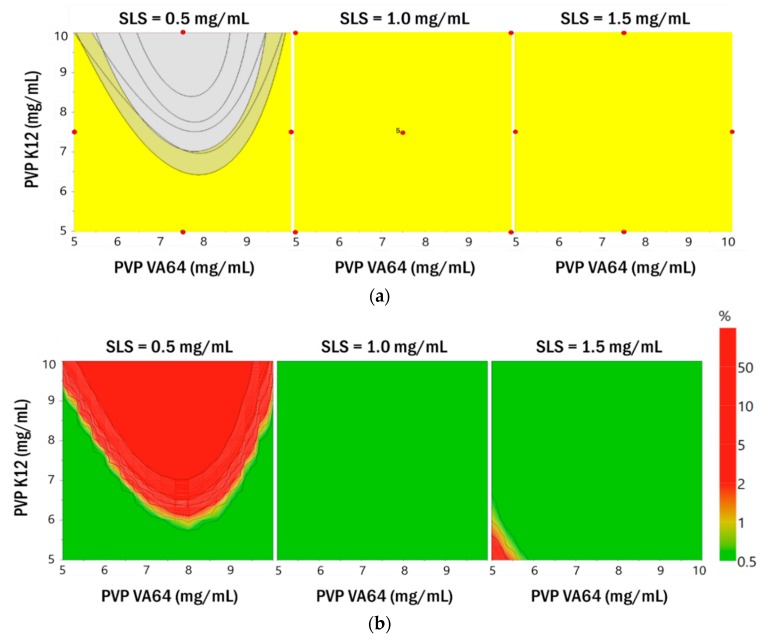
Overlay plots (**a**) and probability maps (**b**) that satisfy the target value for the responses Y_1_–Y_9._

ICH guideline Q8 defines the design space as “the multidimensional combination and interaction of input parameters (e.g., material attributes) and process parameters that have been demonstrated to provide assurance of quality”. Work within this design space is not considered a change, and a flexible and robust process can be established. In this study, the target values were set to <250 nm (Y_1_–Y_4_), <1 μm (Y_5_–Y_8_), and >±20 mV (Y_9_) and a 95% confidence interval was applied. Overlay plots were constructed by including all the responses (Figure 5). The yellow area in the plots indicates the area that satisfies the target value. The established yellow region satisfied the target response value in all regions when the concentrations of SLS were 1.0 mg/mL and 1.5 mg/mL. However, the area of yellow was reduced when the concentration of SLS was 0.5 mg/mL. As shown in Figure 5, probability maps were drawn using MODDE software, based on the calculated model of particle size and zeta potential and the Monte Carlo simulation for RA [37]. Monte Carlo simulations were performed for error propagation for the function relating the independent variables to the dependent variables, giving distributions of dependent variables (i.e., resolutions) for each operating condition of the response surface methodology [38,39,40].

The design space established by the Monte Carlo simulation is expected to be considerably smaller than that of the overlay plot. However, the area obtained from the Monte Carlo simulation is a robust area that has little error and satisfies the set target value. In this study, the design space was represented by the probability of failure for each combination of parameters. A probability of failure of 1% was selected, identifying the design space as an area that met the defined factor with a probability of 99% or greater for a predicted CQA. For each combination, 10,000 simulations were performed and to account for variability in Monte Carlo simulations, 95% confidence intervals were applied. The design space in the green area shown in Figure 5 is a robust area where reliable experimental results are expected. The established green area satisfied the target response value in all regions when the SLS concentration was 1.0 mg/mL. That is, this area ensures robustness. Therefore, the optimum point was identified in the region where the concentration of SLS was 1.0 mg/mL, to minimize the use of the surfactant. Based on the desirability function, the optimal values of the parameters were obtained by graphical and numerical analyses using Design Expert^®^ 11.0 and the formulation that could produce robust products with the targeted quality characteristics was determined [41,42,43]. The combined formulation (PVP VA64, PVP K12, and SLS) that maximized the desirability function was identified (10.0 mg/mL, 5.0 mg/mL, and 1.0 mg/mL). This combination is expected to achieve the smallest particle size and zeta potential. From the regression model generated in this study, the 95% prediction interval for the response was calculated for the optimized formulation shown in Table S7 (Supporting Information). The Box–Behnken design utilized for the optimization of the formulation parameters could be further employed to calculate the system suitability values for the selected responses. The observed values for the responses were within the 95% prediction interval. Therefore, the validity of the model generated in this study was confirmed.

#### 3.2.3. Robustness of Process Parameters in the Optimized Formulation

**Figure 6:**
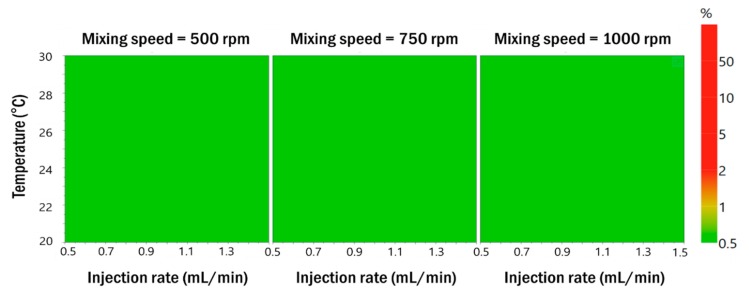
Probability maps that satisfy the target value for the responses when the mixing speed is 500 rpm, 750 rpm, and 1000 rpm in Monte Carlo simulations.

To establish the robustness of the process parameters in the formulation of the optimized resveratrol nanosuspension, nine experiments were performed using a full factorial design and the results are shown in Table 4 (Table S8, Supporting Information). The measured particle size distribution and zeta potential satisfied the target values. The probability map obtained by Monte Carlo simulations showed less than 1% defect probability in all areas (Figure 6). Therefore, the process parameters of the optimized formulation were found to be robust. RA was performed based on experimental data obtained using the Box–Behnken design and the full factorial design. These results are summarized in Table S9 (Supporting Information). The risk level of all formulation and process parameters decreased to low.

### 3.3. Characterization of the Optimized Resveratrol Nanosuspension

#### 3.3.1. Dissolution Characterization

**Figure 7:**
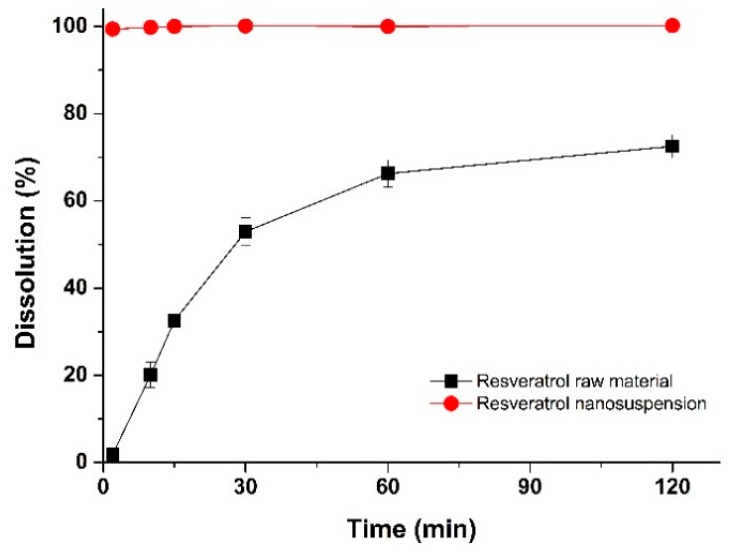
Dissolution profiles of resveratrol in the optimal nanosuspension formulation and the resveratrol raw material.

The in vitro drug dissolution performance of the optimal resveratrol nanosuspension formulation was compared with the drug dissolution performance of the raw material. The dissolution profiles of the resveratrol nanosuspension and the raw material are shown in Figure 7. The dissolution rate was higher for the resveratrol nanosuspension than for the raw material. After 2 min, drug dissolution in the resveratrol nanosuspension and the raw material was 99.4% and 1.9%, respectively, and after 30 min, drug dissolution was 100.1% and 52.9%, respectively (*p* < 0.05). This indicated that the resveratrol nanosuspension had a higher drug dissolution rate than the resveratrol powder.

#### 3.3.2. Pharmacokinetics in Rats

**Figure 8:**
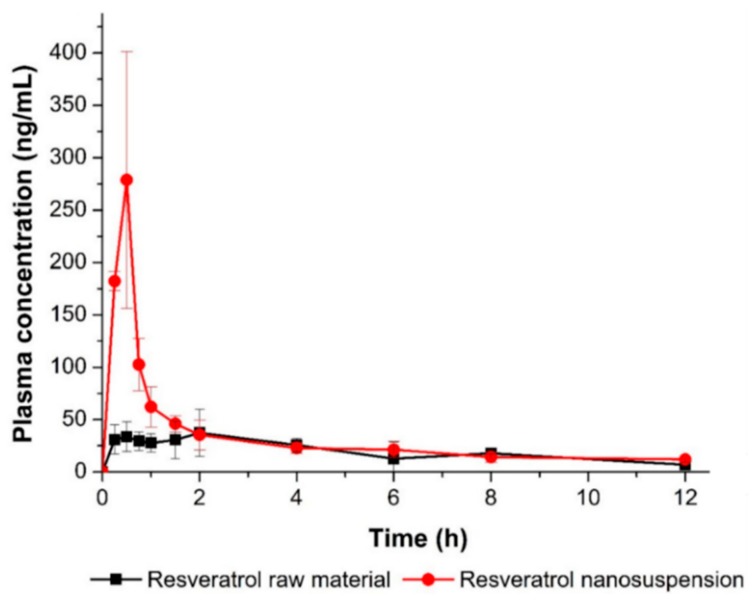
Plasma concentration-time profile of resveratrol in rats after oral administration of the resveratrol nanosuspension and the resveratrol raw material. Data are expressed as the mean ± standard deviation (*n* = 4).

To determine whether the nanosuspension drug delivery system could enhance oral bioavailability, an in vivo experiment was performed using SD rats, to compare the pharmacokinetic parameters of the resveratrol nanosuspension and the resveratrol raw material. Figure 8 shows the plasma concentration-time profile of resveratrol after oral administration of both the nanosuspension and the raw material. The pharmacokinetic parameters (*AUC*_0–12 h_, *C*_max_, and *T*_max_) are presented in Table 6. As shown in Figure 8, the plasma concentration of the resveratrol nanosuspension, with a rapid drug absorption rate, was dramatically higher than that of the resveratrol raw material. The *AUC*_0–12 h_, *C*_max_, and *T*_max_ values of the nanosuspension were 387.0 ± 26.0 ng∙h/mL, 301.4 ± 79.6 ng/mL, and 0.44 ± 0.13 h, respectively. The oral absorption of the resveratrol nanosuspension was higher than that of the resveratrol raw material, with approximately 1.6- and 5.7-fold increases in *AUC*_0–12 h_ and *C*_max_ values, respectively (*p* < 0.05; Table 6). For orally administrated drugs, dissolution is a critical rate-determining step for absorption. When nanosuspensions enter the gastrointestinal tract, nanoparticles provide a larger surface area for the dissolution and molecular dispersion of resveratrol, leading to an increased solubility and concentration gradient on the surface of the nanoparticles, according to the classical passive diffusion theory [15,44]. These results suggested that the oral absorption of resveratrol was significantly increased by its incorporation in a nanosuspension form.

#### 3.3.3. Long-Term Stability

**Figure 9:**
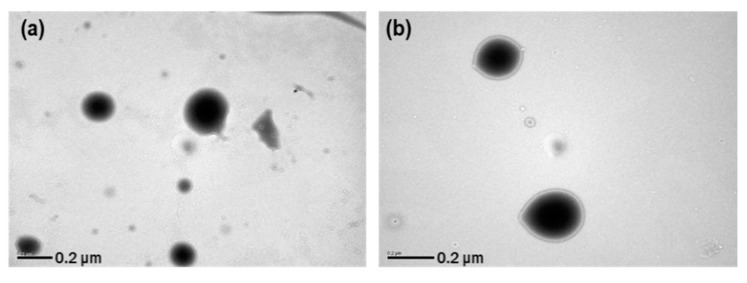

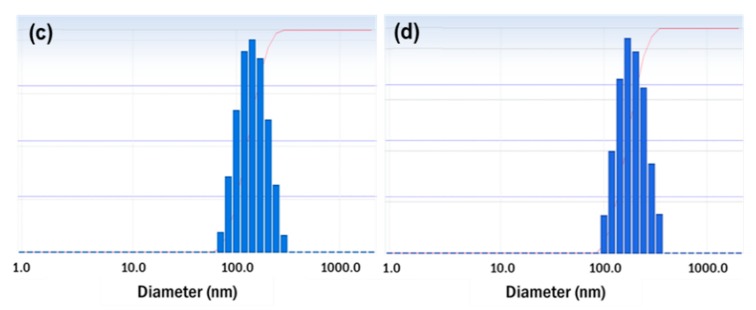
TEM images and particle size distributions of resveratrol nanosuspension. (**a**) Initial (1 day) TEM image, (**b**) 6-month TEM image, (**c**) initial (1 day) particle size distribution, and (**d**) 6-month particle size distribution.

**Figure 10:**
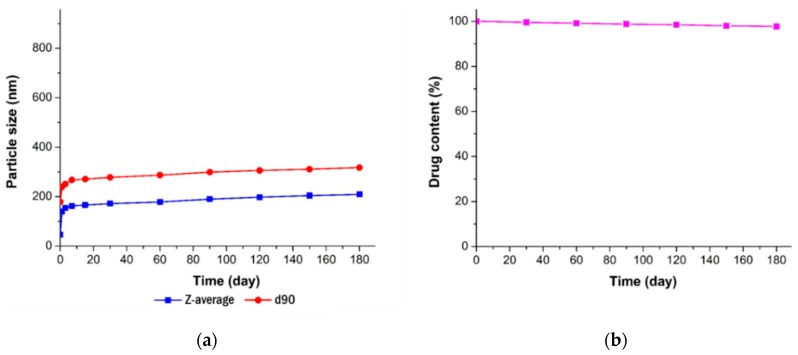
Long-term stability of the optimized resveratrol nanosuspension. (**a**) Particle size (z-average and d90) and (**b**) drug content.

The stability of the resveratrol nanosuspension was analyzed over a 6-month period, including evaluations of particle size, zeta potential, and drug content. As shown in Figure 9, particles generated using antisolvent precipitation were primarily spherical with smooth surfaces and diameters of 46.3 nm at the initial stage of production. The particle size increased dramatically at the early stage of storage after preparation of nanosuspension, then it gradually stabilized over time. This is presumably due to that resveratrol, dissolved in the system as a supersaturated state, might be crystallized at the surface particle, hence induces crystal growth and a particle size increase. In that crystal growth procedure, the primary particle precipitated right after the antisolvent process would have acted as a seed. After 6 months, the particle size was about 200 nm in size and spherical. As shown in Figure 10 (Table S10, supporting information), the particle size showed a value of less than 250 nm, and the drug content was greater than 95%. Accordingly, the nanosuspension stabilized with PVP VA64/PVP K12/SLS does not cause agglomeration or degradation of resveratrol for 6 months, maintaining a physico-chemical state satisfied the target response values [45,46,47].

## 4. Conclusions

A resveratrol nanosuspension with enhanced oral bioavailability was successfully prepared using an antisolvent precipitation method without solvent removal, based on the QbD approach. This study confirmed that the selection of QTTP and CQA, and preliminary studies followed by stepwise RAs helped to efficiently achieve optimization of formulation and process parameter by minimizing trial and error without much useless experiments. First of all, the QTPP was defined and the particle size distribution, zeta potential, and drug content were selected as CQAs to ensure the quality of the resveratrol nanosuspensions. Based on preliminary studies, an updated RA confirmed that the risk levels of the selected formulation parameters were reduced except for the stabilizer concentration. The optimum nanosuspension composition was obtained using a three-factor, three-level Box–Behnken design and a full factorial design confirmed the robustness of the used antisolvent process over the used process parameters range. The dissolution rate and in vivo absorption of nanosuspension optimized by QbD approach were found to be greater than those of the resveratrol raw material. Therefore, it can be concluded that the preparation of a resveratrol nanosuspension, by applying the QbD approach, may be an effective strategy for the development of a new dosage form of resveratrol, with enhanced oral bioavailability.

## Figures and Tables

**Table 1:** Quality target product profile (QTPP) and critical quality attribute (CQA) elements of the resveratrol nanosuspension.

QTTP Elements	Target	Justification
Dosage form	Nanosuspension	Small particles in the nanosuspension increase bioavailability by improving solubility and the dissolution rate [18].
Route of administration	Oral	Oral administration is familiar to patients and results in high compliance with the medication.
Dosage strength	100 mg (5 mg/mL or 10 mg/mL)	The usual dose of resveratrol is 100 mg.
Stability	Long-term stability for 2 years (at least 6 months)	This is the minimum period to confirm the stability of the final product.
Pharmacokinetics	Higher *AUC*_0–12 h_ and *C*_max_, with lower *T*_max_	Improved bioavailability and therapeutic efficacy are achieved through increased drug absorption compared to suspension of resveratrol raw material.
CQA elements	Target	Justification
Particle size (z-average)	<250 nm	Commercial nano-formulation products have particle sizes <400 nm. To develop a product superior to a conventional commercial product, a particle size <250 nm is desired.
Particle size (d90 *)	<1 μm	All particle sizes of the nanosuspension should be <1 μm.
Zeta potential	>±20 mV	To disperse the particles, a repulsive force must be maintained on the particle surface. The desirable zeta potential value corresponding to electrostatic and steric stabilization interactions is >±20 mV [24].
Drug content	95.0–105.0%	The drug content has a high effect on safety and efficacy.

* d90 represents the point of the size distribution ‘containing’ 90% of the total material volume of the sample.

**Table 2:** Summary of risk assessment for resveratrol nanosuspension after preliminary studies.

CQAs	Initial Risk Level			Updated Risk Level		
	Particle Size	Zeta Potential	Drug Content	Particle Size	Zeta Potential	Drug Content
Resveratrol concentration	High	Low	Low	Low	Low	Low
Stabilizer type	High	High	Low	Low	Low	Low
Stabilizer concentration	High	High	Low	High	High	Low
Solvent type	High	Low	Low	Low	Low	Low
Ratio of solvent/anti-solvent	High	Medium	Low	Low	Low	Low
Mixing speed	High	Low	Low	Medium	Low	Low
Mixing time	High	Low	Low	Low	Low	Low
Injection rate (solvent)	High	Low	Low	Medium	Low	Low
Temperature	High	Low	Low	Medium	Low	Low

Low: Broadly acceptable risk. No further investigation is needed. Medium: Risk is acceptable. Further investigation may be needed to reduce the risk. High: Risk is unacceptable. Further investigation is needed to reduce the risk.

**Table 3:** Variables in the Box–Behnken design.

Formulation Variables	Level Used		
	−1	0	1
X_1_ = Concentration of PVP VA64 (mg/mL)	5	7.5	10
X_2_ = Concentration of PVP K12 (mg/mL)	5	7.5	30
X_3_ = Concentration of SLS (mg/mL)	0.5	1	1.5
Response variables	Goal		
Y_1_ = particle size (z-average, initial)	<250 nm		
Y_2_ = particle size (z-average, 1 day)	<250 nm		
Y_3_ = particle size (z-average, 3 days)	<250 nm		
Y_4_ = particle size (z-average, 7 days)	<250 nm		
Y_5_ = particle size (d90, initial)	<1 µm		
Y_6_ = particle size (d90, 1 day)	<1 µm		
Y_7_ = particle size (d90, 3 days)	<1 µm		
Y_8_ = particle size (d90, 7 days)	<1 µm		
Y_9_ = zeta potential (ζ)	>±20 mV		

**Table 4:** Box–Behnken design and full factorial design matrix representing experimental runs with parameters and responses.

StandardOrder	Parameters			Responses								Zeta Potential (mV)
	X_1_	X_2_	X_3_	Particle Size (Z-Average, nm)				Particle Size (d90, nm)				
				Initial	1 Days	3 Days	7 Days	Initial	1 Days	3 Days	7 Days	
Box–Behnken design												
1	5.0	5.0	1.0	108.1	147.7	163.8	171.0	186.4	238.5	254.3	283.2	−45.12
2	10.0	5.0	1.0	46.5	138.9	155.2	170.4	158.8	246.9	250.1	257.5	−38.02
3	5.0	10.0	1.0	139.3	191.7	210.8	223.0	220.5	290.4	335.3	340.7	−39.02
4	10.0	10.0	1.0	44.7	147.7	171.7	186.2	179.3	264.2	289.6	291.4	−30.63
5	5.0	7.5	0.5	165.5	191.6	208.5	208.5	256.0	320.8	336.6	324.3	−32.70
6	10.0	7.5	0.5	64.4	216.2	228.9	228.3	186.3	331.3	355.7	320.4	−34.66
7	5.0	7.5	1.5	87.0	155.1	169.6	177.6	157.9	235.1	262.6	263.8	−42.55
8	10.0	7.5	1.5	33.7	101.7	130.7	145.2	184.0	237.2	262.2	258.0	−35.57
9	7.5	5.0	0.5	127.0	217.1	230.8	233.8	225.5	328.8	333.8	339.5	−41.36
10	7.5	10.0	0.5	155.5	257.7	274.8	270.8	269.0	373.0	426.8	408.0	−32.44
11	7.5	5.0	1.5	68.7	153.4	168.5	175.1	174.0	247.7	263.9	266.5	−41.25
12	7.5	10.0	1.5	69.9	166.1	184.3	198.1	185.3	274.3	297.6	302.8	−35.52
13	7.5	7.5	1.0	85.6	203.2	211.8	220.4	200.6	309.5	344.7	325.6	−38.02
14	7.5	7.5	1.0	84.0	203.5	217.4	217.5	189.1	301.3	327.8	336.5	−42.06
15	7.5	7.5	1.0	77.5	200.3	217.1	215.7	194.8	306.1	326.3	335.9	−38.67
16	7.5	7.5	1.0	82.8	204.2	220.1	221.9	198.6	306.9	331.5	322.2	−39.84
17	7.5	7.5	1.0	76.7	199.3	215.0	216.6	193.7	302.3	336.1	317.9	−37.57
Full factorial design												
1	0.5	20	500	48.0	133.0	151.9	157.8	170.1	245.3	254.2	260.3	−32.97
2	1.5	20	500	53.3	136.6	158.6	163.9	188.0	241.7	265.9	264.3	−36.24
3	0.5	30	500	49.6	136.1	157.3	164.1	184.7	247.7	269.4	265.7	−38.07
4	1.5	30	500	52.7	132.8	152.7	160.4	183.6	241.0	261.7	271.9	−39.07
5	0.5	20	1000	52.4	136.9	156.0	162.7	168.2	232.5	262.0	265.1	−38.02
6	1.5	20	1000	54.7	140.4	157.6	163.5	192.2	245.5	253.8	261.5	−39.60
7	0.5	30	1000	46.0	132.2	156.1	162.0	161.7	236.4	255.5	274.4	−34.59
8	1.5	30	1000	43.1	127.6	152.2	160.2	175.2	245.6	243.8	277.1	−35.91
9	1.0	25	750	49.9	135.2	158.2	165.7	159.2	241.1	256.3	262.3	−36.19

**Table 5:** Summary of results of regression analysis for fitted model of Box–Behnken design.

Response	*R* ^2^	Pred. *R*^2^	PRESS	%CV	*p*-Value	Lack of Fit
Y_1_	0.9907	0.8915	2515.8	6.21	<0.0001	0.1491
Y_2_	0.9959	0.9465	1220.5	2.01	<0.0001	0.0724
Y_3_	0.9911	0.8850	2300.2	2.54	<0.0001	0.0783
Y_4_	0.9858	0.9334	995.7	2.25	<0.0001	0.0869
Y_5_	0.9753	0.7569	3522.4	3.38	<0.0001	0.1300
Y_6_	0.9929	0.9483	1304	1.64	<0.0001	0.1638
Y_7_	0.9726	0.6543	11840.4	3.69	<0.0001	0.0948
Y_8_	0.9503	0.6010	9548.2	3.91	0.0002	0.1357
Y_9_	0.8629	0.0684	234.1	5.47	0.0087	0.3160
Regression equation of the fitted model						
Y_1_ = 81.33 − 38.83X_1_ + 7.38X_2_ − 31.65X_3_ − 8.27X_1_X_2_ + 11.96X_1_X_3_ − 6.82X_2_X_3_ − 7.16X_1_^2^ + 10.48X_2_^2^ + 13.47X_3_^2^.						
Y_2_ = 202.07 − 10.20X_1_ + 13.27X_2_ − 38.28X_3_ − 8.80X_1_X_2_ − 19.50X_1_X_3_ − 6.99X_2_X_3_ − 39.01X_1_^2^ − 6.57X_2_^2^ + 3.07X_3_^2^.						
Y_3_ = 216.29 − 8.29X_1_ + 15.41X_2_ − 36.24X_3_ − 7.63X_1_X_2_ − 14.83X_1_X_3_ − 7.05X_2_X_3_ − 35.54X_1_^2^ − 5.38X_2_^2^ + 3.69X_3_^2^.						
Y_4_ = 218.89 − 6.25X_1_ + 16.00X_2_ − 30.68X_3_ − 9.04X_1_X_2_ − 13.07X_1_X_3_ − 30.11X_1_^2^.						
Y_5_ = 195.36 − 14.06X_1_ + 13.68X_2_ − 29.47X_3_ + 23.94X_1_X_3_ − 8.06X_2_X_3_ − 13.26X_1_^2^ + 4.14X_2_^2^ + 13.93X_3_^2^.						
Y_6_ = 305.23 − 0.64X_1_ + 17.50X_2_ − 44.93X_3_ − 8.66X_1_X_2_ − 4.38X_2_X_3_ − 35.03X_1_^2^ − 10.20X_2_^2^ + 10.90X_3_^2^.						
Y_7_ = 333.28 − 3.89X_1_ + 30.90X_2_ − 45.82X_3_ − 10.38X_1_X_2_ − 4.90X_1_X_3_ − 38.61X_1_^2^ − 12.35X_2_^2^ + 9.61X_3_^2^.						
Y_8_ = 327.93 − 10.70X_1_ + 24.51X_2_ − 37.76X_3_ − 5.92X_1_X_2_ + 1.24X_1_X_3_ − 8.03X_2_X_3_ − 35.11X_1_^2^.						
Y_9_ = -39.28 + 2.56X_1_ + 3.52X_2_ − 1.72X_3_ + 0.32X_1_X_2_ + 2.23X_1_X_3_ − 0.80X_2_X_3_ + 1.15X_1_^2^ + 1.77X_3_^2^.						

*R*^2^, coefficient of determination; PRESS, predicted residual error sum of squares; CV, coefficient of variation.

**Table 6:** Pharmacokinetic parameters of resveratrol nanosuspension in rats.

Formulations	*AUC*_0–12 h_ (ng∙h/mL)	*C*_max_ (ng/mL)	*T*_max_ (h)
Resveratrol raw material	240.3 ± 42.2	52.5 ± 11.7	1.06 ± 0.83
Resveratrol nanosuspension	387.0 ± 26.0 ^a^	301.4 ± 79.6 ^a^	0.44 ± 0.13

^a^*p* < 0.05 versus the resveratrol raw material. *AUC*_0__–12 h_, the area under the plasma concentration versus time curve calculated using the linear trapezoidal method; *C*_max_, the maximum plasma concentration of *trans*-resveratrol; *T*_max_, the time required to reach *C*_max_. Data are expressed as the mean ± standard deviation (*n* = 4).

## Supplementary Materials

The following are available online at https://www.mdpi.com/1999-4923/11/12/688/s1, Table S1: Initial risk assessment of the resveratrol nanosuspension, Table S2: Particle size of resveratrol nanosuspensions prepared using a polymer, Table S3: Particle size of resveratrol nanosuspensions prepared using a polymer/polymer combination, Table S4: Particle size of resveratrol nanosuspensions prepared using various polymer/polymer/surfactant combinations, Table S5: Particle size of resveratrol nanosuspensions prepared using various resveratrol concentrations in Transcutol HP and various ratios of solvent/antisolvent using PVP VA64/PVP K12/SLS (1.0%/0.5%/0.1%, *w*/*v*), Table S6: Updated risk assessment of resveratrol nanosuspension after preformulation and screening study, Table S7: Summary of results of regression analysis for the fitted model of the full factorial design, Table S8: Predicted values (95% prediction interval) for responses (Y_1_–Y_9_), Table S9: Updated risk assessment of resveratrol nanosuspension after optimization study, Table S10: Summary of long-term stability test results for the optimized resveratrol nanosuspension.

## Abbreviations

*AUC*_0–12 h_	the area under the plasma concentration versus time curve calculated using the linear trapezoidal method
BCS	Biopharmaceutical Classification System
*C*_max_	the maximum plasma concentration of *trans*-resveratrol
CPP	critical process parameter
C_s_	saturated solubility
CQA	critical quality attribute
CV	coefficient of variation
d90	the particle size at 90% cumulative volume percentage
DEGEE	diethylene glycol monoethyl ether
DLS	dynamic light scattering
HPC	hydroxypropyl cellulose
HPLC	high-performance liquid chromatography
HPMC	hydroxypropyl methyl cellulose
ICH	International Council for Harmonization of Technical Requirements for Pharmaceuticals for Human Use
PRESS	predicted residual error sum of squares
PVP	polyvinylpyrrolidone
PVP VA	polyvinylpyrrolidone vinyl acetate
*R* ^2^	coefficient of determination
RA	risk assessment
RC	regenerated cellulose
SD	Sprague–Dawley
SLS	sodium lauryl sulfate
TEM	transmission electron microscope
*T*_max_	the time required to reach *C*_max_
USP	United States Pharmacopeia
QbD	quality by design
QTPP	quality target product profile
