# Supplementary material for: Development of a Resveratrol Nanosuspension Using the Antisolvent Precipitation Method without Solvent Removal, Based on a Quality by Design (QbD) Approach
Source: Pharmaceutics. 2019 Dec 17;11(12):688. doi: 10.3390/pharmaceutics11120688 (PMC6955680; doi:10.3390/pharmaceutics11120688)
Supplement: Supplementary file 1 [file pharmaceutics-11-00688-s001.pdf]

pharmaceutics

Supplementary Materials: Development of a Resveratrol Nanosuspension Using the Antisolvent Precipitation Method, Based on a Quality by Design (QbD) Approach

Do-Hoon Kuk, Eun-Sol Ha, Dong-Hyun Ha, Woo-Yong Sim, Seon-Kwang Lee, Ji-Su Jeong, Jeong-Soo Kim, In-hwan Baek, Heejun Park, Du Hyung Choi, Jin-Wook Yoo, Sung-Joo Hwang, and Min-Soo Kim

Table S1. Initial risk assessment of the resveratrol nanosuspension.

CQAs	Parameters	Risk Level	Justification
Particle size (z-average, d90)	Resveratrol concentration	High	If the concentration of resveratrol is excessively high, particles grow very quickly during the manufacturing process. Therefore, the risk level was high.
	Stabilizer type	High	The ability to inhibit particle growth depends on the type of stabilizer. Therefore, the risk level was high.
	Stabilizer concentration	High	An appropriate concentration of stabilizer has an effective ability to inhibit particle growth. Therefore, the risk level was high.
	Solvent type	High	The solvent and anti-solvent should be sufficiently miscible, and the solvent should have a solubilization effect. Therefore, the risk level was high.
	Ratio of solvent/anti-solvent	High	The solubility of resveratrol for the mixture solvents depends on the ratio of the solvent/anti-solvent. The solubility affects particle growth. Therefore, the risk level was high.
	Mixing speed	High	Depending on the mixing speed, the mixing speed of the anti-solvent and solvent vary, and can affect particle growth rate. Therefore, the risk level was high.
	Mixing time	High	Mixing time can affect particle growth. Therefore, the risk level was high.
	Injection rate (solvent)	High	Depending on the rate of injection of solvent, the mixing speed of the anti-solvent and solvent vary, which can affect the particle growth rate. Therefore, the risk level was high.
	Temperature	High	The solubility of resveratrol changes when the temperature of the solvent changes, which can affect particle growth. Therefore, the risk level was high.
Zeta Potential	Resveratrol concentration	Low	The effect of changes in resveratrol concentration on the zeta potential is insignificant. Therefore, the risk level was low.
	Stabilizer type	High	Depending on the type of stabilizer, the surface charge of the particles differs. Therefore, the risk level was high.
	Stabilizer concentration	High	Depending on the concentration of stabilizer, the surface charge of the particles differs. Therefore, the risk level was high.

	Solvent type	Low	The influence of the type of solvent on the surface charge of the particles was insignificant. Therefore, the risk level was low.
	Ratio of solvent/anti-solvent	Medium	Depending on the solvent/anti-solvent ratio, the concentration of the stabilizer in the mixture solvent varies, which affects the surface charge of the particles. Therefore, the risk level was medium.
	Mixing speed	Low	The effect of mixing speed on the zeta potential is insignificant. Therefore, the risk level was low.
	Mixing time	Low	The effect of mixing time on the zeta potential is insignificant. Therefore, the risk level was low.
	Injection rate (solvent)	Low	The effect of injection rate on the zeta potential is insignificant. Therefore, the risk level was low.
	Temperature	Low	The effect of temperature on the zeta potential is insignificant. Therefore, the risk level was low.
Drug content	Resveratrol concentration	Low	Resveratrol is chemically stable when light is blocked. Nanosuspensions were prepared in a space where light was blocked, and the possibility of drug loss during the manufacturing process is insignificant. Therefore, the risk level was low.
	Stabilizer type	Low	
	Stabilizer concentration	Low	
	Solvent type	Low	
	Ratio of solvent/anti-solvent	Low	
	Mixing speed	Low	
	Mixing time	Low	
	Injection rate (solvent)	Low	
	Temperature	Low	

Table S2. Particle size of resveratrol nanosuspensions prepared using a polymer.

Polymer	Concentration (%, w/v)	Particle size (z-average, nm)	Particle size (d50, nm)	Particle size (d90, nm)
PVP K12	2.0	N.D[a]	N.D	N.D
	1.0	N.D	N.D	N.D
	0.5	N.D	N.D	N.D
	0.1	N.D	N.D	N.D
PVP K17	2.0	N.D	N.D	N.D
	1.0	N.D	N.D	N.D
	0.5	N.D	N.D	N.D
	0.1	N.D	N.D	N.D
PVP K25	2.0	N.D	N.D	N.D
	1.0	N.D	N.D	N.D
	0.5	N.D	N.D	N.D
	0.1	N.D	N.D	N.D
PVP K30	2.0	N.D	N.D	N.D
	1.0	N.D	N.D	N.D
	0.5	N.D	N.D	N.D
	0.1	N.D	N.D	N.D
PVP K90	2.0	N.D	N.D	N.D
	1.0	N.D	N.D	N.D
	0.5	N.D	N.D	N.D
	0.1	N.D	N.D	N.D
PVP VA64	2.0	N.D	N.D	N.D
	1.0	N.D	N.D	N.D
	0.5	N.D	N.D	N.D
	0.1	N.D	N.D	N.D

HPMC 3cp	2.0	515[b]	520	6402
	1.0	867[b]	1004	2034
	0.5	1357[b]	1760	3198
	0.1	N.D	N.D	N.D
HPMC 6cp	2.0	331[b]	357	1290
	1.0	618[b]	573	7441
	0.5	978[b]	1008	1646
	0.1	N.D	N.D	N.D
HPMC 15cp	2.0	N.D	N.D	N.D
	1.0	663[b]	820	1756
	0.5	1122[*]	1337	2318
	0.1	N.D	N.D	N.D

[a]N.D indicates that data cannot be measured and there is no data. [b]Precipitation occurs due to particle agglomeration.

Table S3. Particle size of resveratrol nanosuspensions prepared using a polymer/polymer combination.

Polymer / Polymer	Concentration (%, w/v)	Particle size (z-average, nm)	Particle size (d50, nm)	Particle size (d90, nm)
PVP VA64 / PVP K12	2.0 / 1.0	N.D[a]	N.D	N.D
	2.0 / 0.5	N.D	N.D	N.D
	1.0 / 1.0	1151[b]	1058	6760
	0.5	N.D	N.D	N.D
PVP VA64 / PVP K17	2.0 / 1.0	N.D	N.D	N.D
	2.0 / 0.5	N.D	N.D	N.D
	1.0 / 1.0	N.D	N.D	N.D
	1.0 / 0.5	940[b]	877	7053
PVP VA64 / PVP K25	2.0 / 1.0	N.D	N.D	N.D
	2.0 / 0.5	N.D	N.D	N.D
	1.0 / 1.0	1516[b]	1326	2245
	1.0 / 0.5	500[*]	572	5782
PVP VA64 / PVP K30	2.0 / 1.0	18[b]	15	239
	2.0 / 0.5	436[b]	196	7521
	1.0 / 1.0	N.D	N.D	N.D
	1.0 / 0.5	N.D	N.D	N.D
PVP VA64 / PVP K90	2.0 / 1.0	258[b]	50	3681
	2.0 / 0.5	N.D	N.D	N.D
	1.0 / 1.0	948[b]	1173	2339
	1.0 / 0.5	1014[*]	1500	8217
HPMC 6cp / PVP K12	2.0 / 1.0	N.D	N.D	N.D
	2.0 / 0.5	N.D	N.D	N.D
	1.0 / 1.0	N.D	N.D	N.D
	1.0 / 0.5	380[b]	351	2301
HPMC 6cp / PVP K17	2.0 / 1.0	N.D	N.D	N.D
	2.0 / 0.5	N.D	N.D	N.D
	1.0 / 1.0	N.D	N.D	N.D
	1.0 / 0.5	507[b]	451	7239

HPMC 6cp / PVP K25	2.0 / 1.0	N.D	N.D	N.D
	2.0 / 0.5	N.D	N.D	N.D
	1.0 / 1.0	N.D	N.D	N.D
	1.0 / 0.5	N.D	N.D	N.D
HPMC 6cp / PVP K30	2.0 / 1.0	N.D	N.D	N.D
	2.0 / 0.5	N.D	N.D	N.D
	1.0 / 1.0	N.D	N.D	N.D
	1.0 / 0.5	N.D	N.D	N.D
HPMC 6cp / PVP K90	2.0 / 1.0	N.D	N.D	N.D
	2.0 / 0.5	N.D	N.D	N.D
	1.0 / 1.0	N.D	N.D	N.D
	1.0 / 0.5	N.D	N.D	N.D

[a]N.D indicates that data cannot be measured and there is no data. [b]Precipitation occurs due to particle agglomeration.

Table S4. Particle size of resveratrol nanosuspensions prepared using various polymer/polymer/surfactant combinations.

Polymer / Polymer / Surfactant		Concentration (%, w/v)	Particle size (z-average, nm)	Particle size (d50, nm)	Particle size (d90, nm)
PVP VA64 /	PVP K12 / SLS	1.0 / 0.5 / 0.1	46.5	66.8	179.2
PVP VA64 /	PVP K17 / SLS	1.0 / 0.5 / 0.1	59.2	80.5	178.6
PVP VA64 /	PVP K25 / SLS	1.0 / 0.5 / 0.1	66.2	61.3	552.9
PVP VA64 /	PVP K30 / SLS	1.0 / 0.5 / 0.1	57.5	88.0	212.6
PVP VA64 /	PVP K90 / SLS	1.0 / 0.5 / 0.1	72.6	108.3	255.3
HPMC 6cp /	PVP K12 / SLS	1.0 / 0.5 / 0.1	210.4	217.1	516.2
HPMC 6cp /	PVP K17 / SLS	1.0 / 0.5 / 0.1	203.4	205.3	362.6
HPMC 6cp /	PVP K25 / SLS	1.0 / 0.5 / 0.1	236.2	230.5	525.7
HPMC 6cp /	PVP K30 / SLS	1.0 / 0.5 / 0.1	236.8	232.0	548.1
HPMC 6cp /	PVP K90 / SLS	1.0 / 0.5 / 0.1	208.2	209.7	406.6

Table S5. Particle size of resveratrol nanosuspensions prepared using various resveratrol concentrations in Transcutol HP and various ratios of solvent/antisolvent using PVP VA64/PVP K12/SLS (1.0%/0.5%/0.1%, *w*/*v*).

Resveratrol concentration in Transcutol HP	Ratio of solvent/anti-solvent	Resveratrol in nanosuspension	Particle size (z-average, nm)	Particle size (d50, nm)	Particle size (d90, nm)
100 mg/mL	1/9	10 mg/mL	140.3	101.4	1180.9
100 mg/mL	1/19	5 mg/mL	44.7	60.0	165.6
200 mg/mL	1/19	10 mg/mL	1293.5	1792.1	34516.2
200 mg/mL	1/39	5 mg/mL	225.7	117.2	2780.4

Table S6. Updated risk assessment of resveratrol nanosuspension after preformulation and screening study.

CQAs	Parameters	Risk level	Justification
Particle size (z-average, d90)	Resveratrol concentration	Low	In a preliminary experiment, the resveratrol concentration was fixed at 100 mg/mL to satisfy the define QTPP. Therefore, the risk level was reduced to low.
	Stabilizer type	Low	Based on preliminary experiments, PVP VA64, PVP K12, and SLS were selected as stabilizers. Therefore, the risk level was reduced to low.
	Solvent type	Low	Based on preliminary experiments, Transcutol® HP was selected as a solvent. Therefore, the risk level was reduced to low.
	Ratio of solvent/anti-solvent	Low	Based on preliminary experiments, the solvent/anti-solvent ratio was fixed at 1/19 to satisfy the define QTPP. Therefore, the risk level was reduced to low.
	Mixing speed	Medium	In a preliminary experiment, a nanosuspension that satisfied the target values was prepared at a mixing speed of 750 rpm. However, the mixing speed can still affect particle size. Therefore, the risk level was reduced to medium.
	Mixing time	Low	In preliminary experiments, the effect of mixing time on the particle size distribution was insignificant. Therefore, the risk level was reduced to low.
	Injection rate (solvent)	Medium	In a preliminary experiment, a nanosuspension that satisfied the target values was prepared at an injection rate of 1.0 mL/min. However, the injection rate can still affect the particle size. Therefore, the risk level was reduced to medium.
	Temperature	Medium	In a preliminary experiment, a nanosuspension that satisfied the target values was prepared at 25°C. However, the temperature can still affect the particle size. Therefore, the risk level was reduced to medium.

Zeta Potential	Stabilizer type	Low	In preliminary experiments, PVP VA64, PVP K12, and SLS were selected as stabilizers. Therefore, the risk level was reduced to low.
	Stabilizer concentration	High	Depending on the concentration of stabilizer, the surface charge of the particles is different. Therefore, the risk level was high.
	Ratio of solvent/anti-solvent	Low	In preliminary experiments, the solvent/anti-solvent ratio was fixed at 1/19 to satisfy the define QTPP. Therefore, the risk level was reduced to low.

Table S7. Summary of results of regression analysis for the fitted model of the full factorial design.

Response	R^2	Pred. R^2	PRESS	%CV	p-value	Remark
Y_1	0.9901	0.4876	58.36	1.50	0.0295	Significant
Y_2	0.9912	0.9271	7.70	0.41	0.0028	Significant
Y_3	0.7011	0.1557	47.78	1.18	0.0882	Not significant
Y_4	0.4176	−0.5642	73.12	1.44	0.4009	Not significant
Y_5	0.3285	−0.0400	1166.64	5.90	0.1067	Not significant
Y_6	0.9959	0.9602	7.60	0.26	0.0122	Significant
Y_7	0.3503	−0.1103	517.28	2.55	0.0932	Not significant
Y_8	0.7269	0.4196	168.17	1.36	0.0204	Significant
Y_9	0.9496	0.7110	10.73	1.86	0.0074	Significant

Regression equation of the fitted model

$$Y_1 = 49.97 + 0.97X_1 - 2.12X_2 - 0.93X_3 - 0.92X_1X_2 - 1.12X_1X_3 - 2.39X_2X_3$$

$$Y_2 = 134.54 - 0.10X_1 - 2.28X_2 - 0.16X_3 - 1.85X_1X_2 - 2.10X_2X_3$$

$$Y_3 = 155.63 - 0.03X_1 - 0.72X_2 - 2.11X_1X_2$$

$$Y_4 = 162.25 + 0.17X_1 - 0.15X_2 - 1.55X_1X_2$$

$$Y_5 = 175.88 + 6.79X_1$$

$$Y_6 = 241.87 + 1.50X_1 + 0.71X_2 - 1.96X_3 - 0.85X_1X_2 + 4.05X_1X_3 + 0.30X_2X_3$$

$$Y_7 = 258.09 - 4.52X_3$$

$$Y_8 = 266.96 + 4.74X_2 + 1.97X_3$$

$$Y_9 = -36.74 - 0.90X_1 - 0.10X_2 - 0.22X_3 + 1.88X_2X_3$$

R^2, coefficient of determination; PRESS, predicted residual error sum of squares; CV, coefficient of variation.

Table S8. Predicted values (95% prediction interval) for responses (Y_1–Y_9).

Response	95% PI (low)	95% PI (high)	Predicted mean	Actual mean
Y_1	35.4	58.1	46.7	46.3
Y_2	134.3	149.3	141.8	139.2
Y_3	149.0	169.6	159.3	154.6
Y_4	167.5	183.7	175.6	169.7
Y_5	147.6	169.4	158.5	157.7
Y_6	241.1	260.0	250.5	255.0
Y_7	234.2	281.6	257.9	252.9
Y_8	240.3	286.8	263.5	250.9
Y_9	−43.37	-35.45	−39.41	−38.02

PI, prediction interval.

Table S9. Updated risk assessment of resveratrol nanosuspension after optimization study.

CQAs	Parameters	Risk level	Justification
Particle size (z-average, d90)	Resveratrol concentration	Low	Based on a preliminary experiment, the resveratrol concentration was fixed at 100 mg/mL. Therefore, the risk level was reduced to low.
	Stabilizer type	Low	In preliminary experiments, PVP VA64, PVP K12, and SLS were selected as stabilizers. Therefore, the risk level was reduced to low.
	Stabilizer concentration	Low	The particle size distribution in the optimized nanosuspension satisfies the set target range. Therefore, the risk level was reduced to low.
	Solvent type	Low	Based on preliminary experiments, Transcutol® HP was selected as a solvent. Therefore, the risk level was reduced to low.
	Ratio of solvent/anti-solvent	Low	Based on preliminary experiments, the ratio of solvent/anti-solvent was fixed at 1/19. Therefore, the risk level was reduced to low.
	Mixing speed	Low	The particle size distribution of the nanosuspension prepared at 500 rpm–1000 rpm was within the set target range. Therefore, the risk level was reduced to low.
	Mixing time	Low	Based on preliminary experiments, the effect of mixing time on the particle size distribution was insignificant. Therefore, the risk level was reduced to low.
	Injection rate (solvent)	Low	The particle size distribution of the nanosuspension prepared at an injection rate of 1.0 mL/min was within the set target range. Therefore, the risk level was reduced to low.

	Temperature	Low	The particle size distribution of the nanosuspension prepared at 20°C ~ 30°C was within the set target range. Therefore, the risk level was reduced to low.
Zeta Potential	Resveratrol concentration	Low	The effect of changes in the resveratrol concentration on the zeta potential is insignificant. Therefore, the risk level was low.
	Stabilizer type	Low	Based on preliminary experiments, PVP VA64, PVP K12, and SLS were selected as stabilizers. Therefore, the risk level was reduced to low.
	Stabilizer concentration	Low	In the optimized nanosuspension, the zeta potential value was -32.9 mV to -39.6 mV and satisfied the target range. Therefore, the risk level was reduced to low.
	Solvent type	Low	The influence of the type of solvent on the surface charge of the particles was insignificant. Therefore, the risk was low.
	Ratio of solvent/anti-solvent	Low	Based on preliminary experiments, the solvent/anti-solvent ratio was fixed at 1/19. Therefore, the risk was reduced to low.
	Mixing speed	Low	The effect of mixing speed on the zeta potential was insignificant. Therefore, the risk was low.
	Mixing time	Low	The effect of mixing time on the zeta potential was insignificant. Therefore, the risk was low.
	Injection rate (solvent)	Low	The effect of injection rate on the zeta potential was insignificant. Therefore, the risk was low.
	Temperature	Low	The effect of temperature on the zeta potential was insignificant. Therefore, the risk was low.
Drug content	Resveratrol concentration	Low	Resveratrol is chemically stable when light is blocked. Nanosuspensions were prepared in a space where light is blocked, and the possibility of drug loss during the manufacturing process is insignificant. Therefore, the risk level was reduced to low.
	Stabilizer type	Low	
	Stabilizer concentration	Low	
	Solvent type	Low	
	Ratio of solvent/anti-solvent	Low	
	Mixing speed	Low	
	Mixing time	Low	
	Injection rate (solvent)	Low	
	Temperature	Low	

Table S10. Summary of long-term stability test results for the optimized resveratrol nanosuspension.

Days	Response			
	Particle size (z-average, nm)	Particle size (d90, nm)	Zeta potential (mV)	Drug content (%)
0	46.3	157.7	−38.02	100.02
1	139.2	255.0	N.D[a]	N.D[a]
3	154.6	252.9	N.D[a]	N.D
7	169.7	250.9	N.D[a]	N.D
30	178.7	302.9	−37.61	99.62
60	189.8	311.3	N.D[a]	99.21
90	197.9	312.1	N.D[a]	98.87
120	204.4	311.1	N.D[a]	98.55
150	209.4	320.7	N.D[a]	98.14
180	212.6	321.0	−37.29	97.81

[a]N.D indicates that data cannot be measured and there is no data.
